# Covid-19 in Israel: socio-demographic characteristics of first wave morbidity in Jewish and Arab communities

**DOI:** 10.1186/s12939-020-01269-2

**Published:** 2020-09-09

**Authors:** Daphna Birenbaum-Carmeli, Judith Chassida

**Affiliations:** 1 Faculty of Social Welfare and Health Sciences, University of Haifa, Haifa, Israel; 2 Department of Sociology and Anthropology, Tel Aviv University, Tel Aviv, Israel

**Keywords:** Covid-19, Morbidity rate, Israel, Socioeconomic status, Population density, Elderly population, Internet access, Minority, Jewish, Arab

## Abstract

**Background:**

The first wave of the Covid-19 pandemic hit Israel in late February 2020. The present study examines patterns of the first wave of Covid-19 morbidity in Israel at the macro level, during the period of late February to early June 2020, when the first wave has faded out. The analysis focuses on the significance of four sociodemographic variables: socioeconomic status, population density, rate of elderly population and minority status (Jewish / Arab identity) of the population in cities with 5000 residents or more. Additionally, we take a closer look into the association between morbidity rates and one SES component – home Internet access.

**Methods:**

The article is a cross sectional study of morbidity rates, investigated on a residential community basis. Following the descriptive statistics, we move on to present multivariate analysis to explore associations between these variables and Covid-19 morbidity in Israel.

**Results:**

Both the descriptive statistics and regressions show morbidity rates to be positively associated with population density. Socioeconomic status as well as the size of elderly population were both significantly related to morbidity, but only in Jewish communities. Interestingly, the association was inverse in both cases. i.e., the higher the SES the lower the morbidity and the larger the elderly population, the lower the community’s morbidity. Another interesting result is that overall, morbidity rates in Jewish cities were consistently higher than in Arab communities.

**Conclusions:**

We attribute the low morbidity rates in communities with relatively small elderly populations to the exceptionally high fertility rates in ultra-orthodox communities that sustained increased rates of morbidity; the lower morbidity in Arab communities is attributed to several factors, including the spatial Jewish-Arab segregation.

## Introduction

Israel’s first case of Covid-19 was diagnosed on February 26, 2020. On March 14, 2020, 200 cases were confirmed and additional 40–60 cases were being diagnosed daily. Two days later, on March 16, a general lockdown was declared. Mask wearing in public became obligatory on April 12th, but the requirement was hardly enforced for long weeks thereafter. Professionals offered polarized forecasts, ranging from a total of 10 deaths throughout the epidemic, to ten thousand deaths, accompanied by fatal shortage of ICU facilities, ventilators and medical staff. The first wave climaxed in early April with nearly 10,000 active cases and 500–700 additional confirmed cases a day. Since then, the spread started to slow down. Starting April 19, lockdown restrictions were gradually relaxed. As of early June 2020, Israel had roughly 2000 active cases, less than 300 deaths and a fatality rate of 1.67%, substantially lower than that of most West European countries and well below the world average of 6.15%.^1^ At the time of this writing, a second wave of Covid-19 is plaguing Israel. The present study explores morbidity rates in Israel on a residential community basis, aiming to identify associations with main sociodemographic variables, as they evolved under the lockdown practice.

Scholars have suggested numerous variables that may affect the spread and scope of the pandemic, including medical resources, health coverage, life expectancy, employment rates, income inequality, levels of social connectedness and education, alongside religious affiliation, household size, population age composition, population density and ecological footprint [1]. Of this wide array, the present study focuses on four variables that have been repeatedly suggested in Israel’s public discourse, as influencing local morbidity rates: population density, socioeconomic status (SES), percentage of elderly population and minority status. We supplement these four variables by zooming in on one component of the SES index, that of household internet access, which was pointed out as associated with morbidity in Israel, due to inter-sector differences in access. We start with descriptive statistics of these variables. To the best of our knowledge, despite repeated references in the media and public discourse, no systematic analysis of these variables has been conducted to date. A subsequent multivariate analysis seeks to explain the associations between these variables and morbidity rates.

**Socioeconomic status** has often been mentioned in conjunction with rates of Covid-19 morbidity. In the UK, for instance, the disease appears to have spread along class lines. In the early stages of the outbreak, regions with higher SES were over-represented among the sick. Explanations attributed the excess to more frequent traveling and social contacts, alongside better access to testing [2]. Later on, however, infection rates started to rise in densely populated areas, especially those suffering higher levels of deprivation, affecting primarily people from lower socioeconomic urban areas, many of whom also belonged to ethnic minorities [3]. A similar picture emerged in New York City, where low-income neighborhoods were disproportionately hit by the pandemic [4] The contribution of SES to morbidity rates is thought to operate through various channels of disadvantage, such as posing hurdles to social distancing due to crowded housing, jobs that require physical presence and reliance on public transport for job attendance.^2^

**Population density** is another factor that has long been connected to epidemics. In the present pandemic, high morbidity was documented in dense European and American metropolises like London [3], Paris [5], Milan (https://www.citypopulation.de/en/italy/covid/) and New York [6]. All these cities stood out in comparison to their less heavily populated surroundings, with substantially elevated rates of morbidity. Population density is, however, not the sole determinant of morbidity rates, as powerfully demonstrated by the low incidence of disease in Asian megacities like Hong Kong, Singapore, Seoul and Tokyo.

**Age composition** has been singled out in the present pandemic, as associated with morbidity rates [7]. High mortality was attributed to the presence of numerous elderly people, defining such age structure as creating vulnerability to Covid-19 and especially, to its complications. Social settings in which multi-generation households are prevalent have also been mentioned as more susceptible to contagion and subsequent death, primarily among older people [8].

**Minority status** is yet another factor suggested as associated with increased morbidity. In England, for instance, a high proportion of black and minority residents was found to be the strongest predictor of high Covid-19 death rate and people of ethnic minority descent were over-represented among Covid-19 victims [9]. Evidence in the same direction came from the U.S., where black and indigenous people sustained twice or more Covid-19 deaths per capita as compared to white Americans [10].

In the following, we explore the extent to which these factors – SES, population density, size of elderly population and minority status – were associated with morbidity rates in Israel’s residential communities.

### The research field

Israel’s population currently includes over 9 million people, roughly three quarters of whom are Jewish, 21% - Arabs and 5% family members of Jewish immigrants or persons of Jewish descent who were not recognized by the state as Jewish. The population is almost entirely (97.6%) literate and its life expectancy is 82.5 years [11], among the world’s highest. Israel is highly urban, though its largest cities, Jerusalem and Tel Aviv, count roughly a million and half a million residents respectively. The population density is, however, extremely high, exceeding 410 people per square kilometer, ranking among the densest OECD countries. (The respective figures in the UK, Spain and the USA are 275, 93 and 26.). Israel’s population has been consistently growing since the establishment of the state, due to immigration and an exceptional fertility rate: 3.17 children per Jewish woman and 3.04 per Arab woman [12]. Notable within these overall figures is the Jewish ultra-orthodox subpopulation, which accounts for some 12% of the country’s population, residing in segregated communities. Women’s fertility rate in these communities is 7.1 children per woman and [13].

Arab citizens comprise an ethnic national minority in Israel. Despite formal equality, Arabs in Israel suffer various forms of socioeconomic discrimination and disadvantage. Similar to findings from other settings (e.g., [14, 15]), in Israel, too, the health status of Arab citizens is inferior to that of the Jewish majority, manifesting, among other things, higher infant mortality and shorter life expectancy [12]. The differences are attributed mainly to higher rates of congenital disorders, road accidents and smoking. Later on in life, chronic diseases, especially diabetes and obesity are more prevalent. Structural disparities exert great influence as well, as evident in gaps in access to healthcare, health promotion initiatives and disease detection [16]. Arab communities are highly segregated and even in the nine mixed cities, Jews and Arabs live in separate streets and neighborhoods [17]. Though educational accomplishments of Arab men and women in Israel have risen greatly, the percentage of high school graduates who can be admitted to higher education is lower in the Arab sector than among Jewish Israelis and the rate of university graduates is roughly half as high [18]. The distribution of communities by SES attests to the extreme disparities: Arab localities comprise 82% of the residential communities in the lowest SES rank (1), 80% in the second (2) and 75% in the third (3), but not a single Arab community is included in the top SES ranks of 7–10 [16]. Israel’s Arab population is relatively young, showing a median age of 22, vs. 31 in the Jewish population. The average ages are 26.24 and 34.00 respectively [16].

Against this backdrop and the local discourse about the association between Covid-19 morbidity and the four scrutinized variables, we set off to explore the actual findings.

## Materials and methods

This is a cross-sectional study, investigating all of Israel’s residential communities with more than 5000 people (i.e., full population rather than sample). It focuses on Israel’s cumulative Covid-19 morbidity figures since the beginning of national registration in February 23, 2020, up until June 2, 2020, when the first wave of the epidemic was considered to have been contained.

### Data source

Data was retrieved from the official publication of *Clalit* Health Services, Israel’s largest HMO, which presented daily updates of Ministry of Health cumulative morbidity figures, by residential community. (The dataset is available in https://www.clalit.co.il/he/your_health/family/Documents/city3105.pdf.) The data includes approximately 197 municipalities in Israel.

To supplement this data, we retrieved, for each community, several figures from Israel’s Central Bureau of Statistics’ publications. The added figures included:

1. Population density in every community, calculated as the number of residents divided by the community’s area of jurisdiction. As such, this is a continuous variable.^3^
2. The communities’ SES, on a 1–10 (the highest) scale as defined by Israel’s Central Bureau of Statistics. This is calculated as continuous variable.^4^
3. Proportion of local population aged 65+, thereafter called ‘elderly population’, calculated as the number of 65+ local residents divided by the community’s total population. This is a continuous variable.^5^
4. Ethnic Nationality as a dichotomous variable, defining ‘Jewish’ and ‘Arab’ communities. This is a dichotomous variable.

The dependent variable was the number of confirmed Covid-19 cases in each community in June 2, 2020, and their rate per thousand residents. This continuous variable may be challenged as it heavily depends on the testing policy at any given day. While acknowledging this limitation, we nonetheless consider the official morbidity rate a foundational indicator of local disease spread for several reasons. Morbidity rate was a major component in official assessments of the local spread of the epidemic and as such, was formative of related state policy. This centrality was enhanced by a report, published at the height of the epidemic first wave by the influential Advisory Team to the Center for National Security, that contended, on the basis of emerging local and global evidence, that the number of asymptomatically ill persons was not very large. In light of this clinical and policy shaping significance, we consider the Ministry of Health’s daily morbidity figures a valuable indicator for tracking the spread of Coronavirus in Israel.

### Data processing

Data is presented in two stages. First, descriptive statistics outline the distribution of the research variables and the average figures. In the second section, a multivariate analysis is carried out, in order to assess the association between each variable and a community’s Covid-19 morbidity. The regression data is then used to estimate the *‘marginal effect’* of each of the study’s variables on local morbidity rates.

## Results

**Fig. 1:**
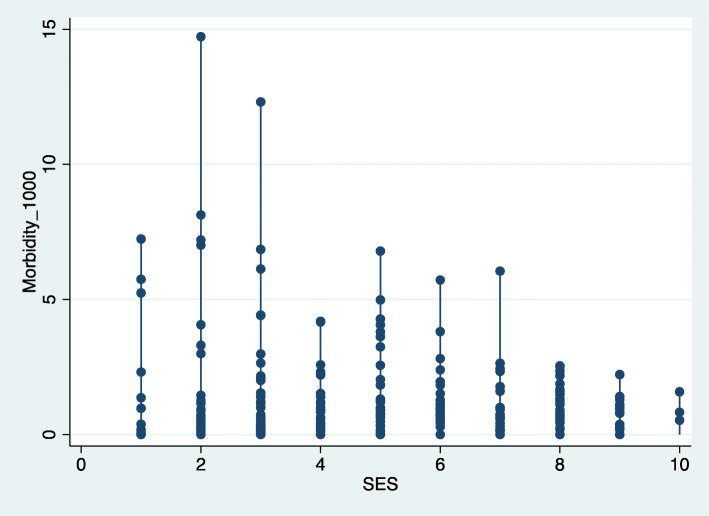
Confirmed morbidity by SES

Looking at Israel’s morbidity rates by SES (Fig. 1), we observe a general trend, suggesting that a rise in SES is associated with a decrease in morbidity rates, i.e., higher morbidity in lower ranking communities and lower morbidity in wealthier ones.

Notably, the SES levels 2 and 3 sustained the highest morbidity rates in Israel. These categories include numerous Jewish orthodox communities (e.g. *Bnei Brak, Elad, Kiryat Yearim, Emanuel, Rechasim* in rank 2 and *Beit Shemesh, and Zefat* in rank 3), which have indeed suffered the highest morbidity in the country. SES level 1 is also populated by orthodox communities. However, it also includes numerous peripheral Arab Bedouin communities. These communities were relatively less affected than communities in the two subsequent levels, as illustrated in Fig. 1. SES category 4 does not include as many orthodox communities, but rather traditional peripheral Jewish as well as Arab settlements. These communities have apparently been relatively less affected than their poorer and wealthier counterparts, possibly due to their peripheral location and distance from major sites of contagion.

**Fig. 2:**
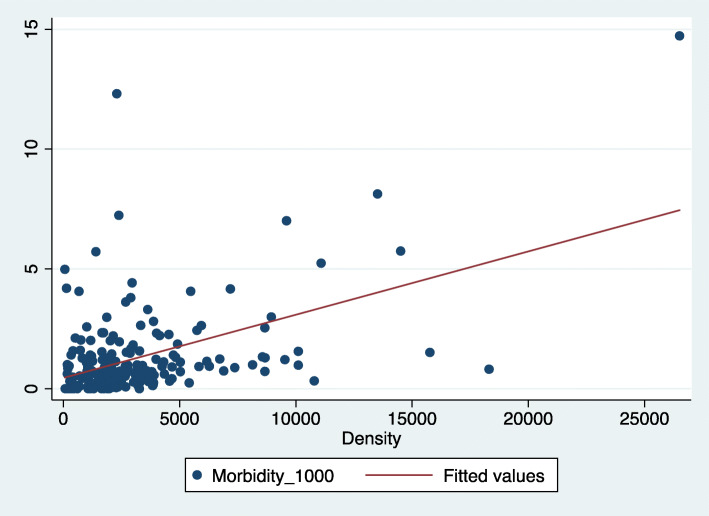
Morbidity Rates by Community Population Density

When assessed by the communities’ population density (Fig. 2), the morbidity distribution appears to follow an even clearer trend, generally increasing with the population density: the higher the density, the higher the morbidity. At the upper right hand corner, the city of *Bnei Brak*, with the highest population density in Israel – roughly 50% denser than the subsequent town (See Table 1) – sustained the highest morbidity rate in Israel during the epidemic’s first wave: 14.72 per 1000 persons. At the upper left corner, the small Arab community of Deer El Assad is an outlier, where morbidity soared for a relatively short period of time in April, but was contained within a few days.

**Table 1:** Covid-19 morbidity rates in Israel’s densest communities

Community	Population density (pop/km^**2)**^	Morbidity (per 1000)
Bnei Brak (O)^a^	26,508	14.72
Givataim	17,589	0.81
Bat Yam	15,651	1.51
Modi’in Ilit (O)	12,953	5.74
Elad (O)	13,515	8.12
Kiriat Motzkin	10,323	0.32
Holon	9882	0.98
Givat Shmuel	9735	1.56
Beitar Ilit (O)	9702	5.23
Ramt Gan	9190	1.21

^a^ Designating an orthodox Jewish community

Nonetheless, as suggested by Table 1, communities differed greatly. For example, though *Elad’s* population density is roughly 3/4 that of *Givataim*, morbidity rate in the former was 10 times that of the latter. Visibly, orthodox cities suffered much greater morbidity than denser non-orthodox communities. Morbidity rates thus increased with population density. Yet, it was clearly not a sole determining factor.

**Fig. 3:**
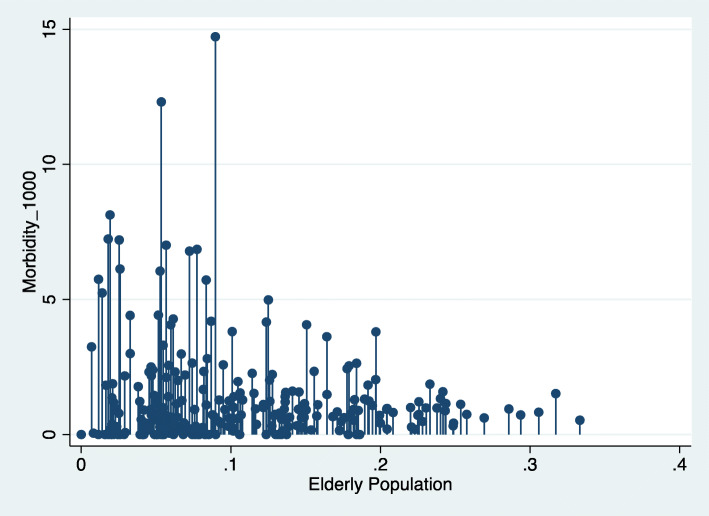
Morbidity rate by number of residents aged 65+

The third factor is the size of elderly population as measured by the proportion of people aged 65+ in the community. The distribution (Fig. 3) depicted an inverse relation between the variables, i.e., the higher the percentage of elderly persons in a community, the lower the morbidity. The two outliers are once again, the communities of Bnei Brak and Deer El Assad, which stood out in the density distribution above (Fig. 2).

The descrpetive statisitics thus suggest 1. Inverse association between a community’s SES and its morbidity rate; 2. Positive association between population density and morbidity; 3. Inverse relation between percentage of elderly population and morbidity.

The last result, which goes somewhat against findings reported from other settings, requires some more attention. We start with the city of *Bnei Brak,* that had the highest morbidity rate in Israel (14.7 per 1000) but a mere 8.9% of elderly residents. Jerusalem and Haifa, in contrast, with evidently larger proportions of elderly residents (12.3 and 27% respectively), had strikingly lower morbidity rates: 4.16 and 0.61 per 1000 respectively. A more systematic look into the communities with the highest morbidity rates (Table 2), shows that the proportions of elderly population in these communities were all below the average for the Jewish sector (13%) and even below the national average (11%), thereby illustrating the inverse association:

**Table 2:** Towns with the highest morbidity rates by percent of elderly population

Town	Morbidity rate (per 1000)	% of population aged 65+
Bnei Brak	14.70	8.9
Elad	8.12	1.9
Kiryat Ye’earim	7.00	5.6
Efrat	5.71	8.3

Notably, all four cities are orthodox Jewish communities.

Exploring the opposite direction, reveals similar findings. As shown in Table 3, the morbidity rates in Israel’s ‘oldest communities’, namely those with the largest proportions of people older than 65 (19-21%), were consistently below (0.32–1.51) the national average of 1.64 per 1000 at the time (June 2, 2020). None of these communities is orthodox.

**Table 3:** Communities with the highest percentage of populate aged 65+

Town	% of population aged 65+	Morbidity rate (per 1,000)	SES
Bat Yam	21	1.51	6
Kiryat Yam	21	0.72	5
Kiriat Motskin	20	0.32	7
Hifa	20	0.61	7
Kiriat Bialik	19	0.41	7

Source: Joint-Israel. 2018. People aged 65+ in Israel. https://brookdale.jdc.org.il/wp-content/uploads/2018/02/MJB-Facts_and_Figures_Elderly-65_in_Israel-2018_Hebrew.pdf

At a glance, the low proportion of elderly people in Israel might be taken as a protective factor that helped moderate morbidity rates in the country. However, the findings suggest the opposite, as illustrated in Tables 2 and 3. The key factor explaining this unusual distribution is the exceptional fertility rates in Israel’s orthodox Jewish communities that stand on 7.1 children per woman, as opposed to roughly 3 children per woman in other Jewish and Arab communities. This difference drastically reduces the percentage of the orthodox communities’ elderly cohorts. At the same time, these very large families are mostly poor, often crowding in small apartments, and as such, are prone to contagion.

In order to gain a closer insight into the association of each variable with morbidity rates, we conducted a linear regression, wherein the dependent variable is the morbidity rate. The following results were obtained (Table 4):

**Table 4:** Linear regression to estimate the association of SES, population density, size of elderly population and ethnicity on morbidity in Israel

Variables	coef. (se)	***β***
SES	−0.122 (0.07)	−0.150
Density	0.00024* (0.00)	0.439
Age 65+	−9.16* (2.28)	−0.343
Jewish / Arab	1.682* (0.39)	0.437
R^2^	0.381	
N	197	

**Fig. 4:**
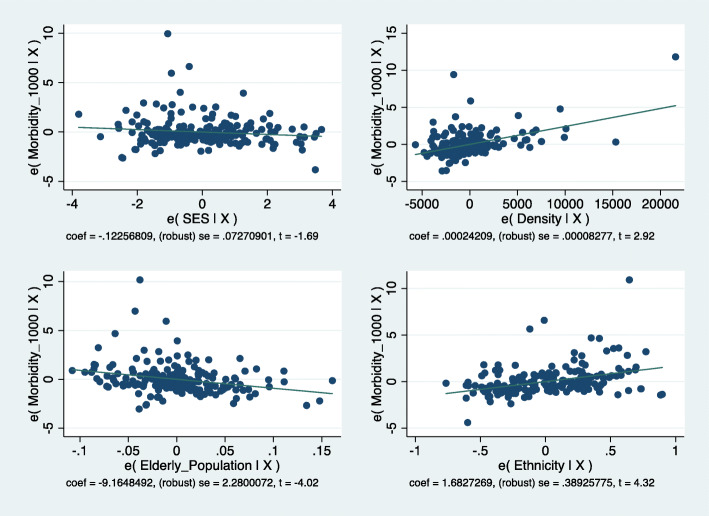
Prediction linear regression of Morbidity in Israel (1:1000)

The regression model thus predicts 38.1% (R^2^ = 0.381) of the inter-community morbidity rate variance in Israel at the community level, by SES, density, elderly population and minority status. As shown, SES turned out to be statistically insignificant, but population density, size of elderly population and minority status emerged as significantly associated with morbidity rates. The association of each variable with COVID-19 communities’ morbidity rates is presented in Fig. 4, which shows the respective regression lines.

**Fig. 5:**
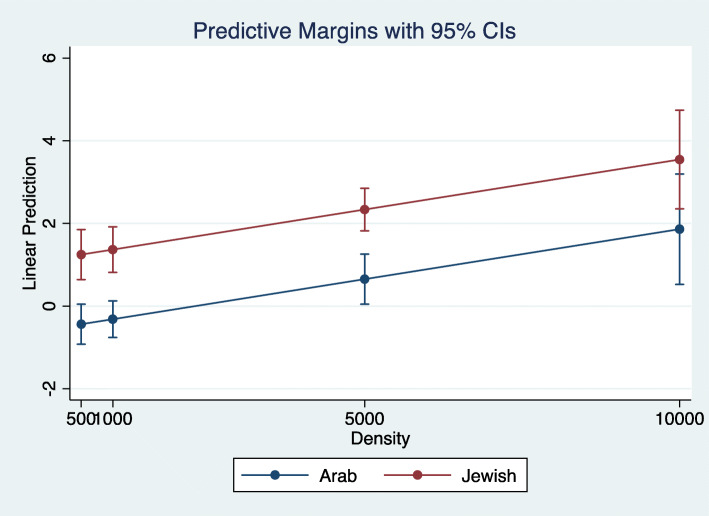
Morbidity in Jewish and Arab cities by community population density

**Fig. 6:**
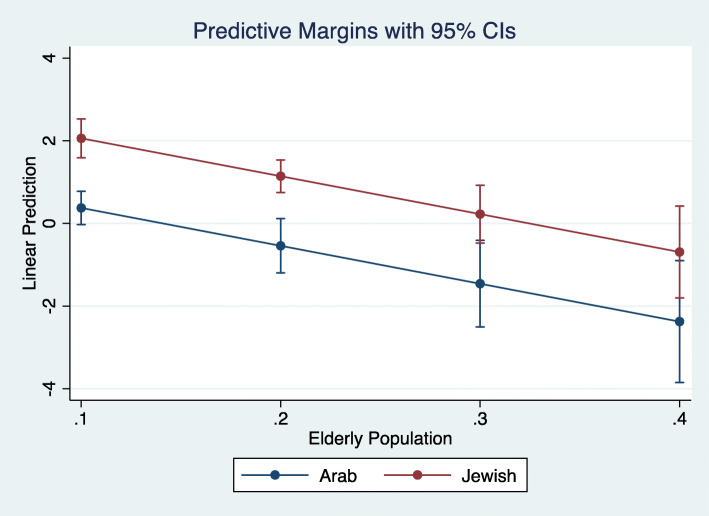
Morbidity rates in Jewish and Arab communities by the number of residents aged 65 +

The nearly horizontal SES line visualizes the lack of statistically significant association between this variable and the rate of morbidity in Israel. Population density, in contradistinction, emerged as significant, affirming the public discourse that linked crowdedness with increased morbidity. Indeed, population density emerged as the strongest association with communities’ morbidity rates (β = 0.439). A 100 person increase per km^2^, raises morbidity by 0.024 patients per thousand (b = 0.00024), namely, 2.4 additional patients per 100,000 persons. As for the percentage of a community’s elderly residents, as suggested by the descriptive statistics, the regression shows a significant inverse association between this variable and communities’ morbidity rates, i.e., the smaller the elderly population, the higher the local rate of morbidity (b = − 9.16). In terms of minority status, despite their lower SES and minority status, Arab communities sustained lower morbidity rates, i.e., Jewish communities suffered 1.68 higher morbidity rates compared to Arab communities (b = 1.68, β = 0.437). Moreover, the Jewish-Arab gap in morbidity proved persistent. Figures 5 and 6 present the marginal association of population density and percent of elderly population – the two variables that were found to be statistically significant – with morbidity rates in the Jewish and Arab populations.

The calculation is based on the regression model, assuming that all other variables are held constant. The figures demonstrate that the higher the population density, the higher the community’s morbidity rates and the larger the elderly population, the lower the morbidity rate. Equally important: at any given value of population density or size of elderly population, the gap in morbidity rate between the Jewish and Arab cities persists, with morbidity rates consistently lower in Arab communities.

In the light of the emergent gap in morbidity between Arab and Jewish communities, we divided our calculation and produced the average values of the scrutinized sociodemographic variables alongside Covid-19 morbidity rates in Jewish vs. Arab communities. Table 5 presents the averge values in each sector.

**Table 5:** Average values of the research variables by communities’ nationality

Average Values	Jewish Communities	Arab Communities
SES	5.77	2.90
Population Density (person/km^2^)	3829	1955
% of residents aged 65+	14.9	6.4
Morbidity Rate (1:1000)	1.64	0.63

The table reveals a clear Jewish-Arab difference: with communities twice or more richer, denser and older, Israel’s Jewish cities sustained 2.6 times the morbidity of their Arab counterparts. Whereas these figures accord with international findings regarding population density and proportion of elderly residents, the higher morbidity in richer cities is exceptional. In other words, Israel’s Arab communities, despite being much poorer and despite belonging to an underprivileged minority, sustained less than half the morbidity of their wealthier Jewish neighbors. Therefore, we examined how these variables are associated with morbidity in Jewish vs. Arab communities. The linear regression analysis presented in Table 6, reveals further details and differences:

**Table 6:** Linear regression model predicting community morbidity rates in Israel

Variables	Non Jewish communities		Jewish communities	
	Model 1	Model 2	Model 1	Model 2
SES	−0.081 (0.13)	−0.046 (0.17)	−0.373* (0.09)	− 0.136 **(**0.07)
Population Density		0.0002* (0.00)		0.0002* **(**0.00)
Persons aged 65+		−5.57 (5.06)		−9.51* **(**2.44)
R^2^	0.78%	10.15%	14.96%	37.46**%**
N	85	71	173	126

The analysis shows that when considered on its own, SES (Model 1) becomes significant in the Jewish communities though not in the Arab ones. Thus, in Israel’s Jewish communities SES was associated with morbidity in a manner similar to that observed elsewhere, i.e., poorer communities were more vulnerable to the virus than wealthier ones. More concretely, a rise in one SES rank in a Jewish community was associated with a reduction of 0.373 sick person per 1000. In the Arab sector, no such association was found. However, once the other variables are added (Model 2), the association of SES with morbidity once again becomes statistically insignificant also in the Jewish sector. Population density (Model 2), however, proved significant and equally influential in both sectors.

The third variable, proportion of elderly population, also emerged as statistically insignificant in the Arab communities but significant and inversely associated with morbidity in Jewish communities, i.e., a smaller elderly population was associated with higher morbidity. Another substantial inter-sectorial difference related to the explained variance. While explaining nearly 38% of the variance in morbidity rates in Jewish communities, Model 2 accounts for less than 11% in Arab settlements. The model thus reaffirmed the lay perception that population density was positively associated with morbidity rates. However, in contrast to international findings, SES was statistically insignificant to morbidity rates in the Arab communities and even in the Jewish sector, the association was weak.

Finally, some Israeli commentators attributed the high morbidity rates in some ultra-orthodox communities to the scarcity of home and mobile Internet access in these communities, that wish to maintain but minimal contact with their non-orthodox surrounding. (‘Kosher mobile phones’ have only pre-designated internet access, e.g., to banks, HMO etc.) This virtual detachment was assumed to underlie the communities’ lack of awareness and belated application of the protective measures and lockdown guidelines. We therefore zoomed in on this specific component of the SES index and conducted a specific linear regression.

The general regression revealed no association with internet access. Sector specific analyses (Table 7) found that home Internet access followed the SES pattern, namely, significant association with morbidity only in Jewish communities and only when considered on its own.

**Table 7:** Linear regression for the prediction of morbidity rate in Israel by household internet access

Variables	Non Jewish communities		Jewish communities	
	Model 1	Model 2	Model 1	Model 2
Households with computer and Internet access (%)	−0.025 (0.01)	−0.019 (0.01)	−0.034* (0.01)	− 0.008 (0.01)
Population density		0.0001* (0.00)		0.0002* (0.00)
Population aged 65+ (%)		−1.54 (4.03)		−9.94* (2.39)
R^2^	11.94%	16.0%	13.48%	43.0%
N	65	65	120	120

However, the latter association was substantial: a 10% increase in home internet access in Jewish communities was associated with a morbidity decline of 0.3 person per 1000. The resemblance to SES patterns is reasonable, given the fact that internet access is a component of the SES index.

**Fig. 7:**
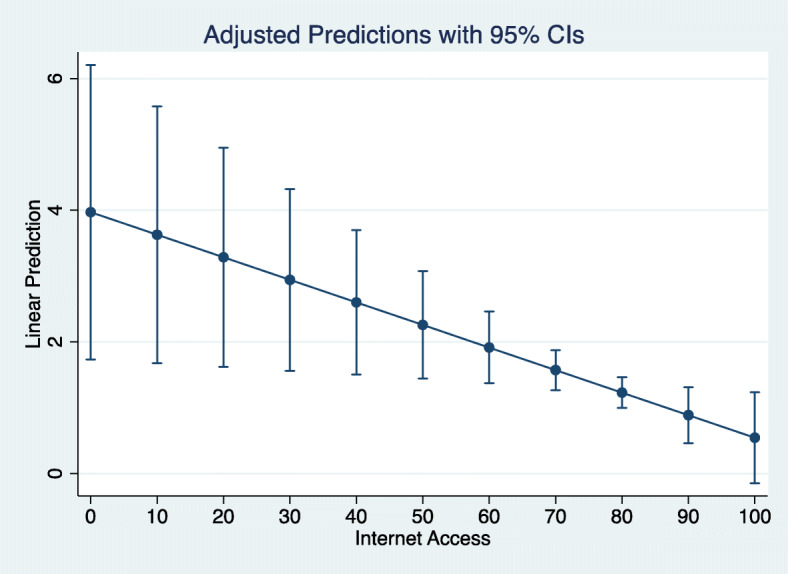
Morbidity in the Jewish sector by community rate of home Internet access

Figure 7 further supports Table 7 findings, showing that as internet access rises, the communities’ morbidity rates decrease.

We now turn to discuss the observed trends and particularities and propose some lines of explanation.

## Discussion

The current analysis revealed increased morbidity rates in dense, low SES Jewish communities with small elderly populations and low access to household Internet. This description befits first and foremost Jewish Orthodox communities. As mentioned, these communities have indeed suffered the highest morbidity rates in Israel. More generally, p**opulation density** was the only variable that emerged as statistically significant to morbidity in the entire Israeli population. In Arab communities, the lower density - 2037 vs. 3853 persons per km^2^ – might have contributed to the lower morbidity. The association of **SES** with communities’ morbidity solely in Jewish cities probably reflects the relatively narrow range of SES distribution of Israel’s Arab communities, which are largely clustered at the lower end of the SES scale. However, in the Jewish sector, too, SES was significant only when analyzed on its own and then, too, the association was moderate: massive differences in morbidity were observed among communities with similar SES^6^ and similar morbidity rates were recorded in communities far apart in their SES ranking.^7^

The association between morbidity and the size of a community’s **elderly population** is probably the most peculiar finding of the present study. Israel’s population is much younger than that of other industrialized countries. The main reason for the gap is the local fertility rate of 3.11 children per woman, which is nearly twice the OECD average of 1.7.^8^ In 2015, people aged 65+ thus comprised 11% of Israel’s population, representing 13% of the Jewish population and a mere 4% of Israeli Arabs. For the sake of comparison, in Japan and Italy the elderly comprise 23 and 28% of the population, respectively.^9^ The OECD average is 17.2%. Having said that, we need to bear in mind that older people do not necessarily contract Covid-19 more often, but are rather at higher risk for complications. As such, the inverse association may not be as paradoxical as it may initially appear to be. In fact, the low proportion of elderly population might have contributed to Israel’s low mortality rate. **I**be. BeyondBeyond this particular difference, the analysis revealed that when density level, SES or size of elderly population are held constant as average, the prediction of morbidity rate in the Jewish communities is 1.68 times higher than in Israel’s Arab communities. This lower morbidity rate among the country’s poor minority population, as compared to the wealthier majority group, counters findings from other countries. Moreover, multi-generation households, another variable associated with higher morbidity, as mentioned, are more prevalent in Israel’s Arab communities than in Jewish ones [19]. How can we explain these surprising findings? What local particularities can account for this difference? We propose the following lines of explanation:

1. High population density and poor, large families might have rendered the lockdown harder to apply in Jewish orthodox communities. It was also harder to self-isolate in the small, congested apartments. The homecoming of children who normally study in religious boarding schools, during the epidemic, further raised the household’s density.
2. For orthodox Jewish men, religious worshiping consists of 2–3 daily gatherings, conducted seven days a week. Possibly, the centrality of this routine made it harder to forego synagogue attendance, especially in the early days of the epidemic, causing delay in adherence and subsequently, excess morbidity. The lower rates of internet access in these communities might have also contributed to the delay in awareness and implementation of the lockdown instructions. Thus, possibly similarly to other places [20], the reduction in social contact was gradual in the orthodox communities, rather than immediate.
3. Numerous orthodox Jews from the U.S. arrived in Israel in the pandemic’s early weeks and for a relatively long period of time were neither tested nor required to self-isolate. In retrospect, many of the incoming people were Covid-19 positive and proved to be a main source of contagion in the orthodox communities [21].

In contradistinction, Israel’s Arab communities, though belonging to an underprivileged, poorer minority (e.g., [22, 23]) and often living in multi-generation households, had several protective factors:

1. Families have fewer children, especially when compared to Ultra-Orthodox Jewish families. Additionally, Arab cities and villages are geographically peripheral and less densely populated.
2. The religious practice of the various Arab religious denominations in Israel – Muslim, Christian and Druze – does not mandate daily gathering. Indeed, Arab religious leaders were early to call for social distancing.
3. Due to various power relations and restrictions that tacitly operate in Israel’s labor market, Arab citizens of Israel, are relatively well represented (though still underrepresented) among the country’s health professionals [24]. Many Arab families thus have a relative who is a nurse, doctor, or pharmacist. These health professionals served, at the outbreak of the epidemic, as educators who spearheaded the new health behavior standards.
4. Having lived as a minority group that is continuously identified as an enemy, Israel’s Arab citizens self-mobilized in collective effort to reduce local contagion. To this end, activists translated into Arabic information material, civil society organizations collaborated and set isolation enabling routines, like extensive collection of donations and distribution of food by volunteers. Probably the most radical practice in this respect was the unprecedented collaboration with the state’s security bodies, which, for the first time in the country’s history, worked together for a common goal [25].
5. Being segregated and often excluded, both national and international ties of Israeli Arabs are not as tight as those of some Jewish orthodox communities, which are closely connected to U.S. Jewish communities. In fact, most incoming traffic to Israel’s Arab communities consisted of local youths studying in Arab or East European countries that had relatively low Covid-19 morbidity at the time.
6. More broadly, the sharp spatial segregation between Arab and Jewish communities in Israel, also means, high ethnic density. As shown elsewhere, this structural feature often coincides with disadvantages like lower access to health care [26] and compromised health status [27], but also with improved mental [28] and physical health [29] and fewer encounters with surrounding racism [30]. Apparently, under the extreme circumstances of the epidemic, Arab communities managed to pull together their resources and coordinate an efficient collective effort to protect themselves. The fear of being stigmatized and blamed for spreading the disease, might have been another motivating factor to the implementation of strict self-discipline [25].

As of May 20, 2020, there were a total of 1040 confirmed Covid-19 cases and five deaths in Arab communities in Israel, since the outbreak of the epidemic [25].

To sum up, Arab communities in Israel were relatively less affected by the Covid-19 epidemic than Jewish communities, despite their relative poverty, minority status and prevalent multi-generational households. Lower population density and smaller families, alongside highly efficient community self-organization and collaboration with state bodies resulted in exceptionally low morbidity and mortality rates. In contradistinction, high morbidity was observed in Israel’s poor, crowded Orthodox communities, especially ones with very small elderly populations.

The observed trends and differences may possibly offer an insight about potential state measures and the protection and inclusion that they have offered to marginalized communities in the context of pandemics. A comparison with morbidity patterns during Israel’s second wave of Covid-19 morbidity may reveal how different containment measures have affected the health and lives of various subpopulations across the social ladder.

### Research limitations

The first limitation of the current study is the focus on confirmed cases. As noted, this figure closely depends on the prevalence of testing. Moreover, assessments of the proportion of asymptomatic ill people vary widely from eight to 54% (e.g., [31]). Additionally, we do not have at our disposal precise data regarding testing in the earlier days of the epidemic, let alone its distribution by communities. Another limitation emerges from the focus solely on the four scrutinized variables. Therefore potentially relevant variables, like education, religiosity, economic disparity have not been addressed. Additionally, since the analysis has been carried out, in June 2020, Israel has been suffering a much greater wave of morbidity. Not only the morbidity rates and spread pattern but also the related policies have changed dramatically. The present analysis thus brackets in only part of the epidemic in Israel. Subsequent comparative analyses that will look into the two waves will be instructive for a more nuanced understanding of the ties between morbidity, policy and social structure, namely, show how various subpopulations are affected by particular policy measures rather than others.

## Footnotes

1 https://ourworldindata.org/mortality-risk-covid; identical to extrapolations from https://www.worldometers.info/coronavirus/, on May 28, 2020.

2 http://info.primarycare.hms.harvard.edu/blog/social-conditions-shape-covid

3 https://www.cbs.gov.il/he/pages/default.aspx

4 https://www.cbs.gov.il/he/publications/doclib/1999/local_authorities/pdf/t02.pdf

5 https://data.gov.il/dataset/residents_in_israel_by_communities_and_age_groups/resource/64edd0ee-3d5d-43ce-8562-c336c24dbc1f

6 e.g., Beitar Illit and Segev Shalom, both rank 1, i.e., at the bottom of the SES scale, but had 4.67 vs. 0 cases per 1000 local residents respectively; Or Yehuda and Or Akiva, both ranked 5 on the SES scale but had 3.76 vs. 0.09 cases per thousand residents

7 Rosh Pina and Segev Shalom had no Covid-19 cases at all, but ranked 1 and 8 respectively; Ganei Tikvah and Netivot had each 2.4 cases per thousand, but ranked 3 vs. 8 on the SES scale.

8 https://data.oecd.org/pop/fertility-rates.htm

9 https://data.oecd.org/pop/elderly-population.htm#indicator-chart
